# Cultural and social drivers of human genetic variation

**DOI:** 10.1002/ctm2.70883

**Published:** 2026-10-23

**Authors:** Alexander Platt, Sarah A. Tishkoff

**Affiliations:** ^1^ Department of Genetics Perelman School of Medicine University of Pennsylvania Philadelphia Pennsylvania USA; ^2^ Department of Biology School of Arts and Sciences University of Pennsylvania Philadelphia Pennsylvania USA

1

Human genetic variation, and its interaction with both environmental and social/cultural factors, strongly influences our health and clinical risks. Before any of this happens, however, the distribution of available variants we find around the globe has itself been shaped by the evolutionary history of our species (Figure 1). To some extent, this evolutionary history reflects the direct functional fitness effects of the genetic variants themselves as, for example, when they aid (or detract from) our ability to extract nutrients from our environment or fend off diseases. Critically, however, the distribution of genetic variation we carry in our genomes has also been shaped by the cultural and social forces of our evolutionary history. Social behaviours, mate choice, and regional cultural practices have all been powerful factors contributing to the modern human gene pool.

**FIGURE 1:**
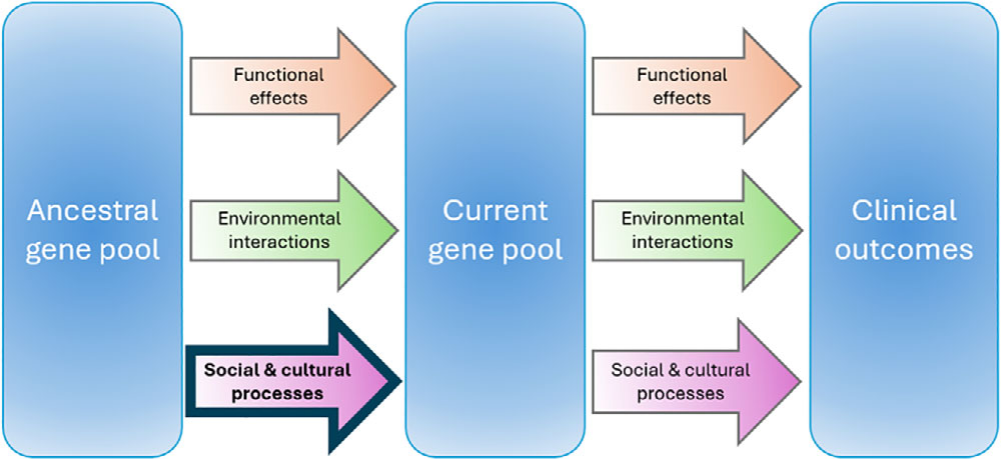
Schematic illustrating multiple ways social and cultural processes influence clinical outcomes. This editorial highlights that, in addition to their roles in mediating how genetic variation manifests in clinical outcomes, (as indicated in bold) social and cultural processes influence the standing distribution of genetic variants available to influence clinical outcomes through both direct and indirect means.

A particularly notable example is the genetic composition of the modern human X chromosome. Anatomically modern humans originated in Africa ∼300 000 years ago. The primary expansion out of Africa ∼50 000 years ago led to interbreeding with Neanderthals in Eurasia (Figure 2). Outside of sub‐Saharan Africa, all modern humans are descended from admixture between Neanderthals and early anatomically modern humans starting ∼45 000 years ago to 40 000 years ago.^1^, ^2^ While a few distinct regions of our genomes (often referred to as ‘Neanderthal deserts’) have subsequently been purged of Neanderthal alleles (likely due to incompatibilities between Neanderthal variants in these regions and modern human variants elsewhere in the genome^3^), we have broadly retained Neanderthal alleles across most of our genome. The large exception is our X chromosomes: Neanderthal X chromosome alleles are almost entirely absent from modern human genomes.

**FIGURE 2:**
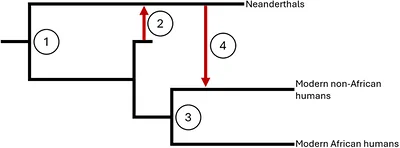
Schematic illustrating general relationship between Neanderthals and modern humans. (1) Neanderthal and modern human lineages diverging ∼600 000 years ago, (2) gene flow from early modern humans into Neanderthals ∼250 000 years ago, (3) expansion of some modern humans out of Africa ∼50 000 years ago, and (4) gene flow from Neanderthals to non‐African humans ∼45 000 years ago.

Broadly, there have been two classes of hypotheses to explain this phenomenon. The first class invokes a direct fitness effect of Neanderthal X chromosome alleles in modern human populations. It contends that individuals carrying more Neanderthal X chromosome alleles were less fit than those with fewer Neanderthal X chromosome alleles, and that the individuals with the fewest Neanderthal X chromosome alleles ultimately out‐competed those with more Neanderthal X alleles for the opportunity to become the direct ancestors of the humans alive today. The assertion in this case is that the Neanderthal X chromosome alleles themselves led to this fitness disparity, either through an accumulation of deleterious variation within the Neanderthal population that was not present in early anatomically modern humans, or through genomic incompatibilities arising between Neanderthal X chromosome variation and modern human genetic backgrounds.

The second class of hypotheses is that the lack of Neanderthal X chromosome alleles in the modern human population has nothing to do with functional variation and is instead a product of the social and cultural relations between Neanderthals and early modern humans. A sex bias in matings between the two groups that favoured pairings between male Neanderthals and female anatomically modern humans would have resulted in relatively fewer Neanderthal X chromosomes entering our gene pool in the first place. With this latter class of hypotheses, the lack of Neanderthal alleles on modern X chromosomes is not the result of natural selection removing Neanderthal X chromosome variation or favouring anatomically modern human X chromosome variation, but reflects the social structure of early human societies, including sex‐specific migration practices and mating preferences.

A recent paper by Platt et al.^4^ finds strong evidence favouring the second class of hypotheses from examining the genomic ‘footprints’ resulting from a different, earlier interaction between Neanderthals and anatomically modern humans ∼250 000 years ago that left early Neanderthals harbouring some ancient anatomically modern human alleles.^3^ All these proposed mechanisms for explaining our modern lack of Neanderthal X chromosome alleles (deleterious Neanderthal alleles, mutual genomic incompatibilities, and sex‐biased interbreeding) make very distinct predictions about where early anatomically modern alleles would be found within Neanderthal genomes.

If, like the autosomal Neanderthal deserts in modern human genomes, the X chromosome is dense with loci where alleles must fit their genomic background (Neanderthal alleles in Neanderthal genomes; modern human alleles in modern human genomes), we would expect to see the same pattern in Neanderthals that we do in modern humans; the same kinds of deleterious interactions would have led to the purging of modern human alleles from their X chromosomes. This did not prove to be the case, however. Neanderthals harbour a larger amount of modern human alleles in their X chromosomes relative to their autosomes.

If instead, Neanderthal X chromosomes were especially replete with deleterious mutations, we would expect that natural selection would favour enrichment of retaining modern human alleles on the Neanderthal X chromosome, *and* that this phenomenon would be most evident in functionally important regions of the chromosomes. While the Neanderthal X chromosome was enriched for modern human alleles on the X chromosome, this phenomenon was primarily driven by intergenic regions and *reduced* in functionally important parts of the chromosome including protein coding sequences, transcription binding sites, and enhancers.

Since the disparity between the amount of modern human alleles Neanderthals retained on their X chromosome and their autosomes was not driven by the functional impact of these genetic variations, it must derive instead from differences in the mode of transmission of genetic material on X chromosomes compared to autosomes. As two out of three X chromosomes in any generation are carried by women, X chromosomes reflect the histories of women more than they do men.^5^ A bias in mating towards pairing of male Neanderthals with female anatomically modern humans can result in an excess of modern human X chromosomes alleles in the Neanderthal genomes and a dearth of Neanderthal X chromosome alleles in contemporary human genomes, without invoking natural selection.

The genomic consequences of sex‐biased demography, social stratification, and migration are not limited to ancient hominin interactions; they represent a continuous feature across ancient and modern human populations. Sex‐biased patterns of migration and dispersal, potentially including a male‐bias in the principle out‐of‐Africa range expansion,^6^ have been instrumental in driving patterns of genetic variation throughout modern humans. Social customs governing mobility, marriage practices, and male‐ or female‐biased migration continue to structure human genetic variation. For clinical and translational researchers, understanding how socio‐cultural evolutionary dynamics have contributed to the composition of the modern gene pool (What variants are present? What variants are common? Who is likely to carry which alleles?) is important for understanding the patterns of genomic variation in modern human genomes and its impact on clinical traits.

## Data Availability

Data sharing not applicable to this article as no datasets were generated or analysed during the current study.
